# Enhancement of NH_3_ Gas Sensitivity at Room Temperature by Carbon Nanotube-Based Sensor Coated with Co Nanoparticles

**DOI:** 10.3390/s130201754

**Published:** 2013-01-30

**Authors:** Lich Quang Nguyen, Pho Quoc Phan, Huyen Ngoc Duong, Chien Duc Nguyen, Lam Huu Nguyen

**Affiliations:** 1 School of Engineering Physics, Hanoi University of Science and Technology, 1 Dai Co Viet Rd, Hanoi 10000, Vietnam; E-Mails: nqlich1501@yahoo.com (L.Q.N.); pq_pho@yahoo.com (P.Q.P.); huyen.duongngoc@hust.edu.vn (H.N.D.); chien.nguyenduc@hust.edu.vn (C.D.N.); 2 Advanced Institute of Science and Technology, Hanoi University of Science and Technology, 1 Dai Co Viet Rd, Hanoi 10000, Vietnam

**Keywords:** multi-walled carbon nanotube, CVD, sensor, NH_3_, catalyst

## Abstract

Multi-walled carbon nanotube (MWCNT) film has been fabricated onto Pt-patterned alumina substrates using the chemical vapor deposition method for NH_3_ gas sensing applications. The MWCNT-based sensor is sensitive to NH_3_ gas at room temperature. Nanoclusters of Co catalysts have been sputtered on the surface of the MWCNT film to enhance gas sensitivity with respect to unfunctionalized CNT films. The gas sensitivity of Co-functionalized MWCNT-based gas sensors is thus significantly improved. The sensor exhibits good repeatability and high selectivity towards NH_3_, compared with alcohol and LPG.

## 1. Introduction

New materials and treatment processes for gas sensing applications that can detect gaseous molecules have attracted increasing attention for several years. Gas sensing studies aim to create an electronic nose that can detect each kind of gas which is present in ambient atmosphere at low concentration levels, with sufficient sensitivity, selectivity, and reproducibility. Several types of gases, such as liquefied petroleum gas (LPG), alcohol, H_2_, NH_3_, NO_2_, and CO_2_, are toxic, harmful, or flammable. Among these gases, NH_3_ is the most common and is used in food processing, environmental remediation, agriculture, and medical diagnostics.

NH_3_ gas sensors have been developed based on semiconductor metal oxides, such as SnO_2_ and ZnO, which require high operational temperatures that range from 300 °C to 400 °C [1,2]. To save energy and reduce the working temperature, researchers are currently looking for new materials to replace these materials. In this aspect, carbon nanotubes (CNTs) hold great promise because of their many advantages. For instance, CNTs function effectively at room temperature and have a high surface per weight ratio. To apply CNTs on NH_3_ sensing application, several gas sensor models based on CNTs, such as gas ionization sensor [3], capacitive sensor [4,5], CNT-field effect transistor [6], resonant-circuit sensor [7], and resistance sensor [8], have been developed. The resistance sensor model is a simpler approach than other models, which are used to fabricate and set up measurement systems.

In this paper, multi-walled carbon nanotube (MWCNT) films were synthesized using the chemical vapor deposition (CVD) method, in which Ni was used as a catalyst on an alumina substrate. The MWCNTs were purified by sintering at 400 °C in dry air. The size and structure of the MWCNTs were observed by field emission scanning electron microscopy (FESEM). NH_3_ sensitivity of the MWCNT films was investigated at room temperature by using a static measurement system. Co-nanoparticle catalyst was coated on the MWCNTs to enhance CNT sensitivity, thereby promoting NH_3_ gas interaction.

## 2. Experimental

In the resistance sensor model, the MWCNT film was synthesized in the area between two Pt electrodes, which were fabricated on an alumina substrate by using a screen printing technique. Before growing the MWCNT film, a Ni catalyst layer was sputtered on this surface. The thickness of the catalyst layer was approximately 8 nm. The substrate was then placed into the reaction chamber. The MWCNT film was synthesized based on the CVD method at 725 °C for 30 min with acetylene (C_2_H_2_) as the gas source. Inert N_2_ gas was used to protect the CNTs from oxygen in the atmosphere. After the growth process, the CNTs were cooled naturally in the reaction chamber, always with the inert N_2_ gas flow.

The purification process was also conducted in the reaction chamber. The sample was heated to 400 °C in dry air to reduce the amorphous carbonaceous material. Oxygen in the atmosphere acts as an oxidizing agent. The morphology of the obtained CNTs was characterized by FESEM. The CNT-based sensor was then used to determine NH_3_ sensitivity by using a static measurement system with a Keithley 6487 picoammeter/voltage source. The gas concentration in the static system was determined using the Canadian BW Gas Alert. A 2 nm thick Co layer was coated on the MWCNT film to enhance the sensing performance of MWCNT to NH_3_ gas. The metal nanoparticle catalyst was used to increase the sensitivity and the selectivity of the CNT-based sensor.

## 3. Results and Discussion

**Figure 1.**
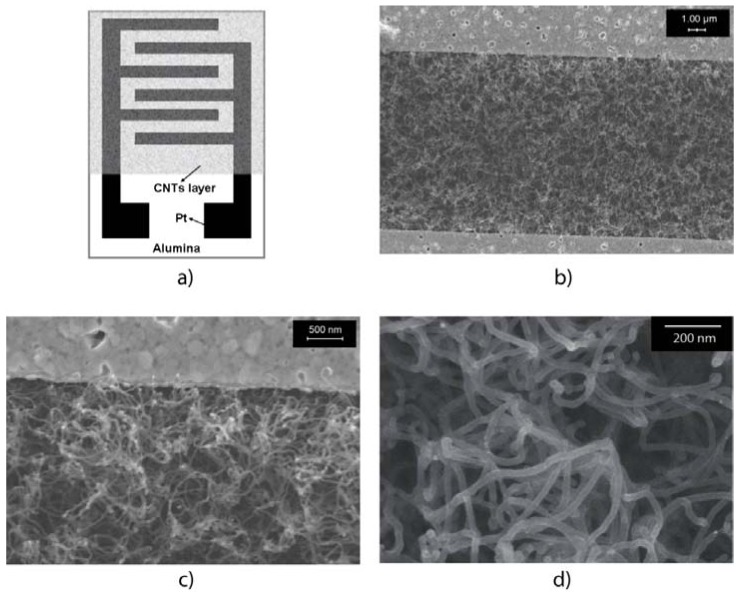
Diagram of the growth of carbon nanotubes (CNTs) on the alumina substrate with Pt electrodes (**a**); CNT formation between two electrodes (**b**); Contact between CNTs and electrode (**c**); CNTs on the alumina surface (**d**).

The diagram of a resistance-type gas sensor is shown in Figure 1(a). The CNT layer was grown on a defined area with two Pt electrodes. Figure 1(b–d) shows the SEM images of the samples synthesized at 725 °C. Before the samples were obtained, the samples were sintered at 400 °C for 30 min in dry air to remove the amorphous carbonaceous material. It is noted that this sintering procedure has been often used to reduce number of impurities and/or defects [9]. The CNTs are very uniform and have an average diameter of approximately 30 nm. The obtained CNTs are multi-walled based on the growth condition and their size, as well as Raman study (not shown here). During the growth process, when the CNTs are long enough, they interact with each others, thereby creating a CNT film, which resembles as a carpet. The FESEM images also show that the CNTs were only formed on the area between the Pt electrodes Figure 1(b). The CNTs are not present in the Pt electrodes because of the difference in the interactions between the metal catalyst and the surfaces.

The CNT growth process mainly occurs through the vapor-liquid-solid mechanism. In this case, CNT growth is supported by catalytic particles and the size of CNT depends on the dimension of these catalytic particles. The catalyst particles play an important role in the growth process. Only suitable catalytic particles can promote CNT formation. Once on the screen printed Pt electrode surface, the catalyst can then penetrate into the Pt electrodes. The Pt layer prevents the metal catalyst atoms from interacting with one another to form suitable particles. In contrast, an alumina surface found in the area between the electrodes helps the catalyst atoms to move and form catalytic particles with suitable sizes. Afterward, CNTs can be grown. The density of CNTs is very high; thus, CNTs can easily bridge the electrodes, thereby creating a contact between the MWCNT film and the electrodes Figure 1(c).

We assumed that the obtained MWCNT behaved as both metallic and semiconducting materials, depending on the synthesis of CNTs, their diameter, chirality as well as the defect and adsorbed gas. Only small amount of semiconducting CNTs formed among predominant conducting CNTs during growing process [10–13]. When the NH_3_ gas adsorbed on the sidewalls, edges or tube ends, the electrical conductivity of the MWCNTs is changed [14,15]. The sensing mechanism of CNT-based gas sensors involves charge transfer, which occurs during the interaction of gas molecules with the CNT surface. This interaction modifies the conductivity of CNTs [8–17]. The resistance of the MWCNT film increases when exposed to NH_3_ molecules, which are electron-donating molecules. This phenomenon likely occurs through the interaction of NH_3_ molecules with the carbon molecules on the CNT sidewalls. Therefore, electrons are transferred from NH_3_ molecules to CNTs. NH_3_ molecules donate electrons to form a space charge region (depletion region) on the semiconducting CNT surface. This depletion region decreases the holes transport, thereby increasing the electrical resistance of CNTs.

**Figure 2.**
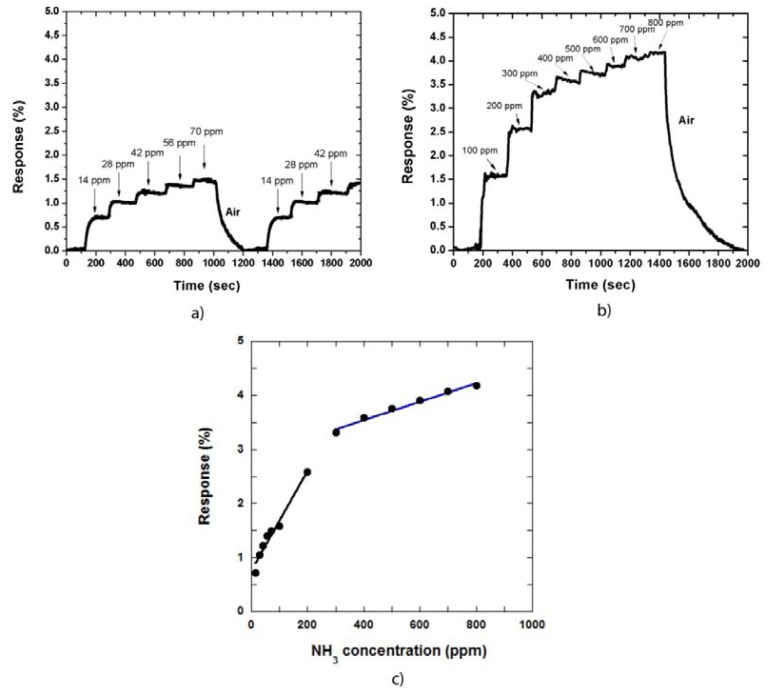
Sensor responses to NH_3_ gas at a low concentration (<100 ppm) (**a**) and high concentration (up to 800 ppm) (**b**). Dependence of the sensor responses on NH_3_ gas concentrations shows two corresponding linear regions (**c**).

In our gas sensing measurement, the sensor response is defined as the relative resistance change: Response (%) = [(*R*_gas_ – *R*_air_)/*R*_air_)] × 100%, where *R*_gas_ and *R*_air_ are the resistance of the MWCNT film in NH_3_ gas environment and in dry air, respectively. Figure 2(a,b) shows the sensor response against the measurement time in low and high NH_3_ gas concentrations, respectively. The CNT sensor responds to NH_3_ at room temperature, *i.e.*, when the NH_3_ gas is adsorbed on the CNT film, the sensor becomes immediately sensitive to the gas. The response then reaches a saturated value at a defined gas concentration.

At low NH_3_ concentrations, the response of the sensor is increased slightly as the gas concentration is increased. For instance, when the gas concentration was increased to 70 ppm, at a step of 14 ppm, the response increased and was maintained at approximately 1.5%. In contrast, the response decreased to approximately 0% when the sensor was exposed to air Figure 2(a). The recovery time in this case was approximately 200 s. The measurements were also repeated by injecting NH_3_ gas into the system at the same concentrations. This result demonstrated that the CNT film was stable during the measurement process. We also determined the sensor response at high NH_3_ gas concentrations of 100 ppm to 800 ppm. Figure 2(b) shows the responses of the CNT film to NH_3_ gas. The response time of the sensor is approximately 30 s to 50 s and the recovery time was approximately 500 s at the NH_3_ gas concentration of 800 ppm.

The relationship between the responses of the sensor and NH_3_ concentration is shown in Figure 2(c), which can be divided into two linear regions: the low concentration region and the high concentration region. The sensitivity increases more rapidly in the low concentration region than that in the high concentration region. Therefore, the fitting curve of the former presented a bigger slope compared with that of the latter. This observation occurs because of CNT film structure, in which the interaction between CNTs and adsorbed NH_3_ gas is increased as gas concentration is increased. However, when the gas concentration is further increased, some NH_3_ molecules are adsorbed on the CNT sidewall, the other molecules must diffuse into the CNT film to find available sites. Therefore, the sensitivity increases slowly in this region.

The CNTs can be functionalized with nanoparticles or composite to improve their gas sensitivity. On the one hand, the functionalization of the CNTs involves the combination of the properties of the CNTs and nanoparticles in nanocomposite CNT/nanoparticle structures, which can exhibit new properties that differ from those of their individual parts. On the other hand, the nanotubes in gas sensing applications are decorated by the nanoparticles to provide selective sensitivity to different gases. Several studies have demonstrated that the CNTs can be decorated with Pd to increase the sensitivity [18], coated with Pt, Pd, Rh, and Au for the selectivity of gas sensor array [19], or sputtered with Ti for gas sensing at low temperature [20]. In this study, we used Co to decorate the obtained CNTs and investigate the effect of Co/CNT interaction on the sensitivity and selectivity of the sensor. The modification of the MWCNTs involves sputtering of ∼2 nm of Co on the entire substrate that contains the MWCNT-based sensor. The CNTs film is coated by very thin Co layer (2 nm). It is revealed that the change in morphology is not clear (the corresponding SEM image is not shown here), except the tubes surface is rougher. The growth of this thin metal layer results in the formation of nanoparticles that decorate the sidewall of the nanotubes [18–20].

**Figure 3.**
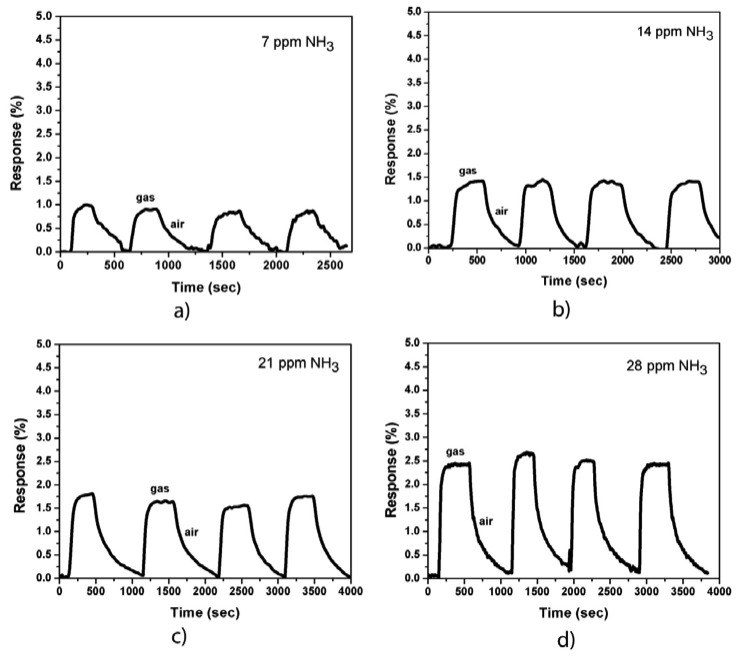
Sensor response to 7 ppm (**a**), 14 ppm (**b**), 21 ppm (**c**), and 28 ppm (**d**) of NH_3_ on and off cycles. The surface of the device is coated with Co (2 nm thick) before gas exposure.

Figure 3 shows the responses of the sensor (MWCNTs were coated with Co nanoparticles) to NH_3_ gas at room temperature and at various gas concentrations (7, 14, 21, and 28 ppm, respectively). The responses of the sensor increased approximately two times compared with that of the uncoated samples. The sensor response cycles against the time of exposure to NH_3_ gas and dry air showed a good repeatability. The response time is approximately 30 s and the recovery time varies from 200 s to 500 s, which depends on the NH_3_ gas concentration. Both the response and recovery times (before and after Co-coating) of our sensors are much shorter compared to some other works [9,13,21]. The increase in the recovery time of Co-coated devices could be explained by the presence of surface chemisorptions additional to physisorption in the case of uncoated samples. Meanwhile, the increase in sensor sensitivity can be caused by the metal/CNT interaction. A depletion region is formed when the CNT sidewall is coated with Co nanoparticles. The NH_3_ molecules donate electrons to the CNT sidewall, thereby expanding the depletion region, which reduces the carrier (holes) transport on the CNT wall and decreases the carrier mobility as well as the conductivity of the device. The sensor resistance is then increased.

**Figure 4.**
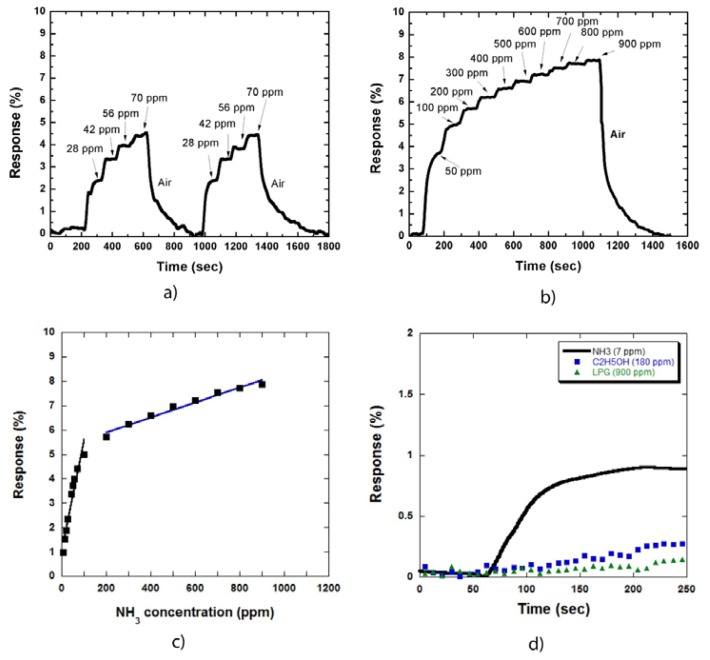
Responses of the MWCNT based sensor decorated with Co nanoparticles to NH_3_ gas at low (**a**) and high (**b**) gas concentration; Dependence of the responses on NH_3_ concentration (**c**); Comparison of the sensor responses to NH_3_, alcohol, and LPG which demonstrated that the sensor was selective to NH_3_ (**d**).

Figure 4(a) shows the two cycles of sensor responses to NH_3_ gas at low concentrations of 28, 42, 56, and 70 ppm (a step of 14 ppm). Figure 4(b) shows the sensor response to NH_3_ gas at high concentrations (up to 900 ppm). The sensor response increased by a factor of two compared with that in Figure 2(a,b). This result is consistent with that in Figure 3, which shows that the increase in response is caused by the expansion of the combined depletion layers of metal/CNTs and NH_3_ molecules/CNTs on the CNT sidewall.

The relationship between the sensor responses and NH_3_ concentration is illustrated in Figure 4(c), which is similar to the result obtained for the samples uncoated with metal nanoparticles Figure 2(c). The responses in the low concentration region increased more rapidly than that in the high concentration region. The decrease in the slope is due to the limit of adsorption sites on the MWCNT sidewall on the sample surface.

Figure 4(d) shows the responses of the sensor to NH_3_ gas compared with alcohol and LPG. NH_3_, alcohol, and LPG concentrations were 7, 180, and 900 ppm, respectively. These concentrations were chosen to observe clearly the different sensor response curves towards these three types of gases. The sensor responded to NH_3_ as soon as the gas was injected. By contrast, the sensor was almost insensitive to alcohol and LPG. The same results were also obtained at higher gas concentrations. Thus, our MWCNT based sensor demonstrated high selectivity to NH_3_ gas at room temperature.

## 4. Conclusions

MWCNTs were successfully grown on the alumina substrate with two Pt electrodes to fabricate a gas sensor. The sensor was sensitive to NH_3_ gas at a wide range of concentrations from some ppm to 900 ppm. Co nanoparticles were decorated on the CNT sidewall to improve both the sensitivity and selectivity of the sensors.
